# Preventing shivering with adjuvant low dose intrathecal meperidine: A meta-analysis of randomized controlled trials with trial sequential analysis

**DOI:** 10.1038/s41598-017-14917-5

**Published:** 2017-11-10

**Authors:** Yu-Cih Lin, Chien-Yu Chen, Yuan-Mei Liao, Alan Hsi-Wen Liao, Pi-Chu Lin, Chuen-Chau Chang

**Affiliations:** 1 Department of Anaesthesiology, Taipei Medical University Hospital, Taipei, 110 Taiwan; 2 School of Nursing, College of Nursing, Taipei Medical University, Taipei, 110 Taiwan; 3 Department of Anaesthesiology, School of Medicine, College of Medicine, Taipei Medical University, Taipei, 110 Taiwan; 4 Graduate Institute of Humanities in Medicine, Taipei Medical University, Taipei, 110 Taiwan; 5 Institute of Clinical Nursing, School of Nursing, National Yang-Ming University, Taipei, 112 Taiwan; 6 Master Program in Long-Term Care, College of Nursing, Taipei Medical University, Taipei, 110 Taiwan

## Abstract

The aim of this systematic review and meta-analysis is to evaluate the pros and cons of adjuvant low dose intrathecal meperidine for spinal anaesthesia. We searched electronic databases for randomized controlled trials using trial sequential analysis (TSA) to evaluate the incidence of reduced rescue analgesics, shivering, pruritus, nausea and vomiting when applying adjuvant intrathecal meperidine. Twenty-eight trials with 2216 patients were included. Adjuvant intrathecal meperidine, 0.05–0.5 mg kg^−1^, significantly reduced incidence of shivering (relative risk, RR, 0.31, 95% confidence interval, CI, 0.24 to 0.40; TSA-adjusted RR, 0.32, 95% CI, 0.25 to 0.41). Intrathecal meperidine also effectively reduced need for intraoperative rescue analgesics (RR, 0.27, 95% CI, 0.12 to 0.64; TSA-adjusted RR, 0.27, 95% CI, 0.08 to 0.91) and the incidence of pruritus was unaffected (RR, 2.31, 95% CI, 0.94 to 5.70; TSA-adjusted RR, 1.42, 95% CI, 0.87 to 2.34). However, nausea and vomiting increased (RR, 1.84, 95% CI, 1.29 to 2.64; TSA-adjusted RR, 1.72, 95% CI, 1.33 to 2.23; RR, 2.23, 95% CI, 1.23 to 4.02; TSA-adjusted RR,1.96, 95% CI, 1.20 to 3.21). Under TSA, these results provided a sufficient level of evidence. In conclusion, adjuvant low dose intrathecal meperidine effectively attenuates spinal anaesthesia-associated shivering and reduces rescue analgesics with residual concerns for the nausea and vomiting.

## Introduction

Occurring in about half of patients receving spinal anaesthesia, shivering^1^ is an undesirable outcome for surgical patients linked to impaired thermoregulatory control^2–4^. Shivering generates heat to maintain body temperature, but it induces stress responses, interferes with surgical procedure and anaesthesia monitoring, and causes postoperative pain and discomfort^5–8^. In addition, perioperative shivering causes lactic acidosis, increases blood, intraocular and intracranial pressure as well as metabolic demand and oxygen consumption^5,9–12^.

Meperidine is an intermediate lipid soluble opioid with potent local anaesthetic properties^13–15^. With dosage of 0.5–1.0 mg kg^−1^ intrathecally for spinal anaesthesia, meperidine provided adequate sensory blockage and postoperative analgesia^15–18^. However, drowsiness and pruritus might occur along with nausea, vomiting and even respiratory depression at such a high dose intrathecal meperidine^19–22^. Due to the above adverse effects, combining local anaesthetics with relatively lower dosage, 0.05–0.5 mg kg^−1^ intrathecal meperidine, were suggested to provide adequate analgesia with less side effects^21–24^.

Although intrathecal meperidine was suggested for both analgesia and shivering prevention in spinal anaesthesia, comprehensive evidence for and against this is lacking^1^. Controversies exist about the dose-dependent anti-shivering effect^25–27^. Intrathecal meperidine, dosage as low as 0.2 mg kg^−1^ or 12.5 mg, was reported to show an anti-shivering effect without nausea and vomiting, but other studies did not show similar effects^27–32^. Controversy remains about intrathecal local anaesthetics combined with lower dose meperidine causing adverse effects such as bradycardia, hypotension, pruritus, nausea and vomiting^21,25–27,31,33^. Therefore, this systematic review and meta-analysis with trial sequential analysis focuses on whether low dose, 0.05–0.5 mg kg^−1^, intrathecal meperidine adjuvant with local anaesthetics could prevent shivering without significant side effects during spinal anaesthsia.

## Materials and Methods

This study was carried out in accordance with the Preferred Reporting Items for Systematic Reviews and Meta-Analyses (PRISMA) guidelines^34^. Review protocol has been registered in PROSPERO (International Prospective Register of Systematic Reviews; number CRD42016051081) before the study.

### Inclusion and exclusion criteria

In duplicate and independently, two reviewers (Y.C.L. and C.Y.C.) screened all articles and abstracts as randomized controlled trials (RCTs) evaluating the effects of low dose, 0.05–0.5 mg kg^−1^ up to 25 mg, adjuvant intrathecal meperidine in patients receiving spinal anaesthesia and noting outcomes of interest (incidence of shivering or drug-related adverse effects). We excluded: 1) interventions delivered through oral, parenteral or epidural routes; 2) different regimens or doses of local anaesthetics given between the experiment and the control; 3) meperidine given alone without local anaesthetics; 4) non-elective surgery; and 5) duplicate reporting of patient cohorts.

### Search strategy and study selection

Y.C.L. and C.Y.C. performed a comprehensive literature search independently in databases including PubMed, EMBASE, the Cochrane Library databases, Google Scholar and the ClinicalTrials.gov registry (http://clinicaltrials.gov/). The relevant keywords used for medical subject headings and free text searches were meperidine, pethidine, demerol, meperidine hydrochloride, spinal anaesthesia, neuraxial anaesthesia, intrathecal anaesthesia, regional anaesthesia, subarachnoid anaesthesia, lumbar anaesthesia, shivering, postoperative shivering, post-anaesthetic shivering, spinal-induced shivering, chills, and chillness. Related citations were used in the PubMed search with no language restrictions through April 2017.

### Data extraction

Two reviewers (Y.C.L. and C.Y.C.) extracted the baseline as well as all outcome data, including the study design, the participant data, the inclusion and exclusion criteria, the type of surgery, temperature control, spinal anaesthetic techniques and drugs adopted, and any resulting complications. If an agreement could not be reached, the dispute was resolved with the help of a third reviewer (C.C.C.). To overcome unit-of-analysis error, we combined groups to create a single pair-wise comparison for the final analysis, and divided the “shared” group equally in subgroup analyses in comparing different regimen of low dose groups, as the Cochrane handbook recommended^35^.

### Methodological quality appraisal

We assessed the quality of each study based on the adequacy of randomization, the allocation concealment, the blinding of the patients and the outcome assessors, the length of the follow-up period, the reporting of study withdrawals, the performance of an intention-to-treat analysis, and other potential bias assessed by Cochrane Collaboration’s tool^36^. Disagreements about subtracted data were adjudicated by a third reviewer (C.C.C.).

### Outcomes and Statistical Analysis

The primary outcome was the incidence and intensity of shivering after receiving spinal anaesthesia. The secondary outcomes included the need for rescue analgesics during the surgery, and related complications such as nausea, vomiting, pruritus, hypotension and bradycardia. We conducted meta-analysis with the Review Manager, version 5.3 (Cochrane Collaboration, Oxford, England), applying trial sequential analysis (TSA) software version 0.9.5.5 beta (http://www.ctu.dk/tsa/)^37^, SAS 9.4 (SAS Institute Inc., Cary, NC, USA) software for meta-regression, and Comprehensive Meta Analysis Version 2 (NJ, USA) for publication bias. A random-effects model was used to calculate the pooled estimates of adjusted relative risk (RR). Standard deviations were estimated from the confidence interval (CI) limit, and the standard error or range values were provided from studies cited. The effect sizes of dichotomous outcomes were reported as RR and the precision value based on a 95% CI. A pooled estimate of value was calculated using the DerSimonian and Laird random-effects model^38^.

To evaluate the statistical heterogeneity and the inconsistency of treatment effects across the studies, the Cochrane *Q* test and *I* ^2^ statistics were used, respectively. Statistical significance was set at 0.10 for the Cochrane *Q* test. The Egger test was used to assess the funnel plot for significant asymmetry, indicating possible publication or other bias^39^. The “trim and fill” method was used to test and adjust publication bias^40,41^.

### Trial sequential analysis (TSA)

TSA combines information size estimation for meta-analysis (cumulated sample size of included trials) with an adjusted threshold for statistical significance in the cumulative meta-analysis which called trial sequential monitoring boundaries^37^. Trial sequential analysis could help us reduce the risk of random error, to increase the robustness of the meta-analyses, and to determine whether the current sample size is sufficiently enough. We applied TSA to the cumulative meta-analysis for all outcomes during spinal anaesthesia^37^. TSA is conducted with an overall 5% of a type I error and a power of 80%. We calculated the alpha-spending adjusted required sample size based on the relative risk reduction (RRR) of each outcome. The cumulative z-curve was constructed using a random-effects model. We set the 95% confidence intervals adjusted for scattered data and repetitive testing.

### Subgroup analysis

For each study, we conducted subgroup analyses by pooling estimates of various surgery types and different dosage adjuvant intrathecal meperidine. Studies of low dose adjuvant intrathecal meperidine was further divided into two groups: dose equal to or less than 0.2 mg kg^−1^ or 12.5 mg (Group I), or dose more than 0.2 mg kg^−1^ or 12.5 mg, up to 0.5 mg kg^−1^ or 25 mg (Group II).

### Meta-regression

We conducted meta-regression to assess the relationship between one or more covariates (moderators) and primary outcome. In this study, potential covariates such as age, meperidine dose, and sample size of each study will be evaluated to determine whether they are the source of heterogeneity.

### Sensitivity analyses

We conducted sensitivity analyses to evaluate methodological quality, participants and spinal anaesthesia techniques to test the stability of the integration effect and to assess statistical heterogeneity. Regarding the quality assessment, bias of selection, performance, detection and attrition were excluded. If random sequence generation and allocation concealment were both unclearly reported or any were categorized as high-risk, we counted this as selection bias.

## Results

### Trial characteristics

**Figure 1:**
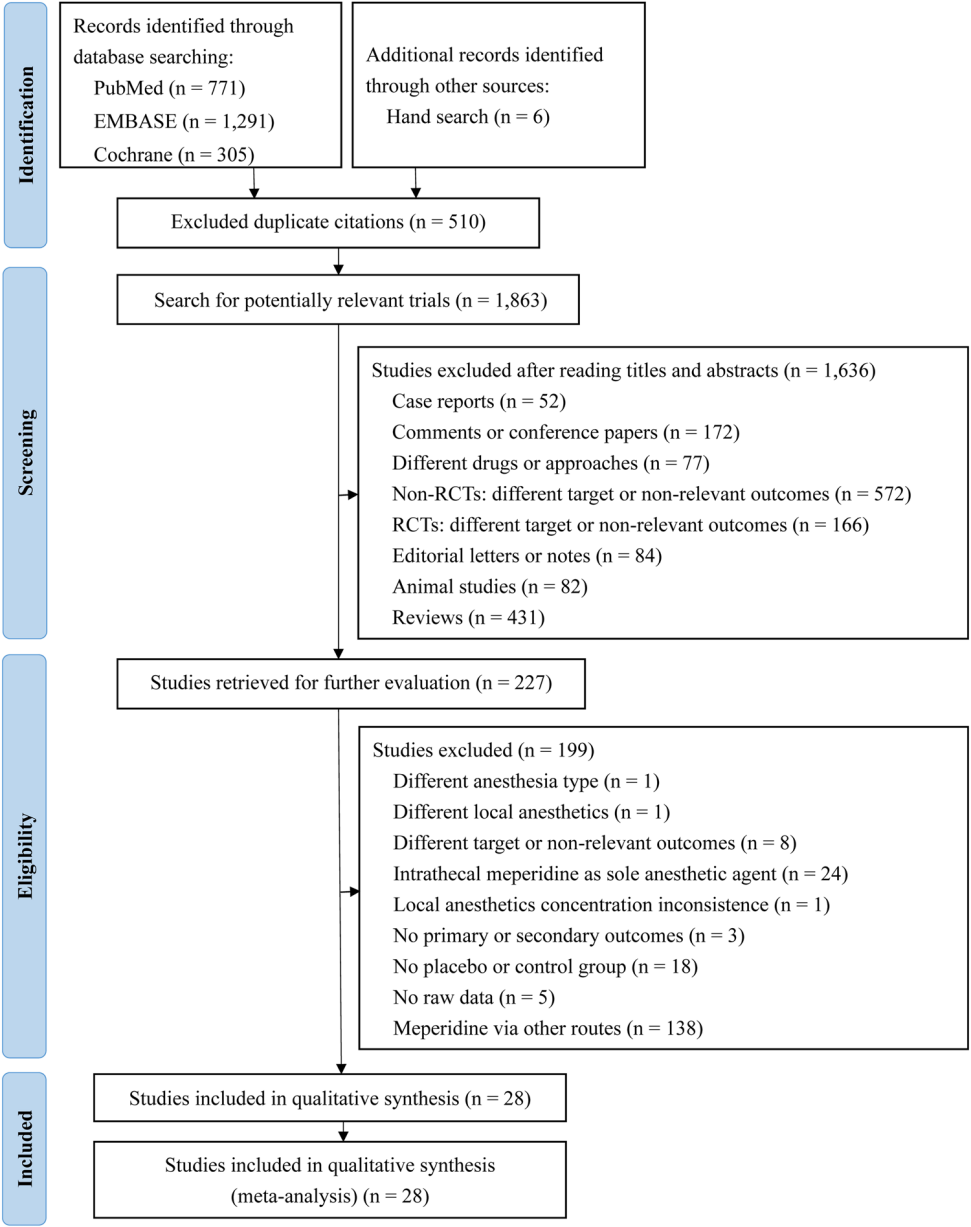
Flowchart for selection of studies by PRISMA flow diagram.

Among 2373 initially evaluated abstracts, 227 studies met the initial inclusion criteria (Fig. 1), 199 trials were subsequently excluded, one study did not use spinal anaesthesia, one did not use the same local anaesthetics, 8 trials had different targets or non-relevant outcomes, 24 studies used intrathecal meperidine as sole anaesthetic agent, one study had inconsistent local anaesthetics concentrations, 3 articles did not have primary or secondary outcomes, 18 trials had no placebo or control groups, 5 trials did not have raw data, and 138 studies used meperidine via other routes. Overall, 28 studies were included in the qualitative synthesis.

Published from1984 to 2016, the characteristics of the 28 RCTs with 2216 participants were shown in Table 1 ^16,25–33,42–59^. Three studies were published in Korean^51,53,56^, two were in traditional Chinese^33,49^, and two were in simplified Chinese^42,43^, one was in Turkish^50^, and the remaining 20 were published in English. The sample sizes ranged from 40 to 195 patients. There are 15 RCTs of obstetric patients receiving caesarean section surgery^25,27–29,31,32,42,43,48,52,53,56–59^, 4 trials enrolled urological surgery^16,44,47,55^, and the other 9 focused on lower abdominal or lower limb surgery^26,30,33,45,46,49–51,54^. Intrathecal local anaesthetics plus meperidine group was compared with the same dose local anaesthetics alone^16,25,28,42,43,49^ or with normal saline^26,27,29–32,44–48,50–59^ or with 10% glucose water^33^ as controls.

Five studies evaluated the anti-shivering effects of intrathecal fentanyl with meperidine^46,50,53,56,58^, three trials interrogated it with intrathecal morphine^28,43,54^ and one with intravenous ondansetron^30^. Two trials added morphine for both the experimented and the controlled^52,57^, while one contained epinephrine^58^. Eight studies investigated various doses of intrathecal meperidine^16,25–27,42,43,53,59^. In terms of the intrathecal local anaesthetic drug, bupivacaine was used in 21 studies^25–28,30–32,42–46,48,50–54,56,57,59^, lidocaine in 5 trials^16,29,47,55,58^, and tetracaine in 2 RCTs^33,49^. Intravenous sedation was combined with spinal anaesthesia in 4 studies^16,49,51,54^, and prehydration was neither given nor disclosed in 4 trials^16,45,51,54^. Ten RCTs did not provide information on hypothermia prevention^31–33,45,47,50,52–54,58^. Patient characteristics, spinal anaesthesia techniques and surgical procedures are summarized in Table 1

**Table 1:** Characteristics of the selected 28 randomized controlled trials

Study (year)	No. of patient (male %)	Age (Mean ± SD)	Type of surgery/ASA	Preoperative medication/prehydration	Intraoperative temperature control: OR/fluid/drapes use	SA technique: position/space/needle size	Intervention: spinal anesthesia drugs
Anaraki and Mirzaei (2012)^25^	C: 39 (0)	28.7 ± 4.9	C/S/I-II	Unclear/37 °C LR 10 mL kg^−1^	21–23 °C/37 °C/drapes	Sitting/L4-5/25 G	C: 0.5% bupivacaine (H) 10 mg
	M_1_: 39 (0)	28.7 ± 4.9					M_1_: 0.5% bupivacaine (H) 10 mg + meperidine 0.2 mg kg^−1^
	M_2_: 39 (0)	28.7 ± 5.0					M_2_: 0.5% bupivacaine (H) 10 mg + meperidine 0.3 mg kg^−1^
	M_3_: 39 (0)	28.7 ± 4.9					M_3_: 0.5% bupivacaine (H) 10 mg + meperidine 0.4 mg kg^−1^
Anaraki, *et al*. (2012)^47^	C: 39 (100)	67.2 ± 8.4	Suprapubic prostatectomy/I-III	NM/NS 10 mL kg^−1^	Unclear/unclear/unclear	Sitting/L4-5/25 G	C: 5% lidocaine (H) 100 mg + NS
	M: 38 (100)	68.1 ± 8.1					M: 5% lidocaine (H) 100 mg + meperidine 0.5 mg kg^−1^
Chen, *et al*. (1993)^49^	C: 30 (67)	36.9 ± 11.8	Surgery of lower limbs or abdomen/I-II	Valium/room air LR	21–22 °C/room air/drapes	Lateral/L3-4/23-25 G	C: tetracaine (H) 12-16 mg
	M: 30 (60)	34.3 ± 10.9					M: tetracaine (H) 12-16 mg + meperidine 0.2 mg kg^−1^
Choi, *et al*. (2000)^53^	C: 11 (0)	29.8 ± 2.1	C/S/I-II	No/LR 500-1,000 mL	Unclear/unclear/unclear	Lateral/L3-4 or L4-5/25 G	C: bupivacaine (H) 9 mg + 1 mL NS (PF)
	F: 11 (0)	28.3 ± 2.5					F: bupivacaine (H) 9 mg + fentanyl (PF) 0.15 μg kg^−1^
	M_1_: 11 (0)	27.7 ± 2.5					M_1_: bupivacaine (H) 9 mg + meperidine (PF) 0.25 mg kg^−1^
	M_2_: 11 (0)	30.5 ± 2.2					M_2_: bupivacaine (H) 9 mg + meperidine (PF) 0.5 mg kg^−1^
Chun, *et al*. (2010)^44^	C: 25 (100)	65.8 ± 7.8	TURP/unclear	Unclear/LR 300-500 mL	24 °C /unclear/ blanket + Bair Hugger	Lateral/L3-4 or L4-5/25 G	C: 0.5% bupivacaine 8 mg + NS
	M: 25 (100)	67.3 ± 7.4					M: 0.5% bupivacaine 8 mg + meperidine (PF) 0.2 mg kg^−1^
Chung, *et al*. (1997)^52^	C: 16 (0)	Unclear	C/S/I-II	Metoclopramide/LR 1,500-2,000 mL	Unclear/unclear/Unclear	Sitting/L2-3 or L3-4/24 G	C: 0.75% bupivacaine 12 mg + morphine 0.15 mg + 0.2 mL NS
	M: 16 (0)						M: 0.75% bupivacaine 12 mg + morphine 0.15 mg + meperidine 10 mg
	M_1_: 17 (0)						M_1_: 0.75% bupivacaine 12 mg + meperidine 10 mg + 0.2 mL NS
Davoudi, *et al*. (2007)^55^	C: 40 (100)	70.0 ± 9.9	TURP/I-III	Unclear/37 °C LR 15 mL kg^−1^	22–25 °C/37 °C/unclear	Sitting/L3-4 or L4-5/25 G	C: 5% lidocaine (H) 75 mg + NS
	M: 40 (100)	72.7 ± 9.3					M: 5% lidocaine (H) 75 mg + meperidine 15 mg
Farzi, *et al*. (2014)^58^	C: 65 (0)	32.2 ± 7.3	C/S/I-II	Unclear/NS 10 mL kg^−1^	Unclear/unclear/unclear	Sitting/L3-4 or L4-5/25 G	C: lidocaine 70 mg + epinephrine 0.1 mg + 0.5 mL NS
	M: 65 (0)	28.6 ± 6.1					M: lidocaine 70 mg + epinephrine 0.1 mg + meperidine 25 mg
	F: 65 (0)	27.7 ± 6.0					F: lidocaine 70 mg + epinephrine 0.1 mg + fentanyl 25 μg
Fidan, *et al*. (2008)^45^	C: 20 (35)	39 ± 11	Unilateral knee arthroscopy/I-III	NM/no	Unclear/unclear/unclear	Lateral/L3-4/27 G	C: bupivacaine (H) 6.5 mg + NS
	M: 20 (25)	43 ± 11					M: bupivacaine (H) 6.5 mg + meperidine 10 mg
Fu and Chang (2008)^43^	C: 30 (0)	28.1 ± 3.4	C/S/I-II	Unclear/23-25 °C Ringer’s 15 mL kg^−1^	21-23 °C/23-25 °C/unclear	Lateral/L2-3/unclear	C: 0.75% bupivacaine 8-10 mg
	M_1_: 30 (0)	27.1 ± 3.5					M_1_: 0.75% bupivacaine 8-10 mg + meperidine 5 mg
	M_2_: 30 (0)	28.0 ± 2.1					M_2_: 0.75% bupivacaine 8-10 mg + meperidine 10 mg
	Mo_1_: 29 (0)	31.1 ± 4.5					Mo_1_: 0.75% bupivacaine 8-10 mg + morphine 0.1 mg
	Mo_2_: 30 (0)	30.5 ± 3.2					Mo_2_: 0.75% Bupivacaine 8-10 mg + morphine 0.2 mg
Han, *et al*. (2007)^56^	C: 20 (0)	31.8 ± 4.0	C/S/I-II	Unclear/HS 15 mL kg^−1^	22-24 °C/unclear/drapes	Unclear/L4-5/26 G	C: 0.5% bupivacaine (H) 8.5 mg + NS
	M: 20 (0)	32.3 ± 4.3					M: 0.5% bupivacaine (H) 8.5 mg + meperidine (PF) 12.5 mg
	F: 20 (0)	33.2 ± 4.3					F: 0.5% bupivacaine (H) 8.5 mg + fentanyl 12.5 μg
Honarmand, *et al*. (2015)^26^	C: 30 (76.7)	Data error	Lower limb orthopedic surgery/I-II	Unclear/37 °C LR 15 mL kg^−1^	21-23 °C/37 °C/unclear	Sitting/L3-4 /25 G	C: 0.5% bupivacaine 15 mg + NS
	M_1_: 30 (80)						M_1_: 0.5% bupivacaine 15 mg + meperidine 0.1 mg kg^−1^
	M_2_: 30 (86.7)						M_2_: 0.5% bupivacaine 15 mg + meperidine 0.2 mg kg^−1^
	M_3_: 30 (80)						M_3_: 0.5% bupivacaine 15 mg + meperidine 0.3 mg kg^−1^
Hong and Lee (2005)^28^	C: 30 (0)	31.3 ± 4.5	C/S/I-II	Unclear/23-25 °C LR 15 mL kg^−1^	23-25 °C/unclear/drapes	Lateral/L3-4/unclear	C: 0.5% bupivacaine 8-10 mg
	M: 30 (0)	30.8 ± 4.3					M: 0.5% bupivacaine 8-10 mg + meperidine 10 mg
	Mo_1_: 29 (0)	30.5 ± 3.2					Mo_1_: 0.5% bupivacaine 8-10 mg + morphine 0.1 mg
	Mo_2_: 30 (0)	29.7 ± 1.8					Mo_2_: 0.5% bupivacaine 8-10 mg + morphine 0.2 mg
Imarengiaye, *et al*. (2011)^31^	C: 25 (0)	30.7 ± 3.9	C/S/I-II	Ranitidine/NS 15 mL kg^−1^	Unclear/unclear/unclear	Sitting/L2-5/25 G	C: 0.5% bupivacaine (H) 10 mg + 0.15 mL NS
	M: 25 (0)	32.2 ± 5.0					M: 0.5% bupivacaine (H) 10 mg + meperidine 7.5 mg
Khan, *et al*. (2011)^27^	C: 24 (0)	27.1 ± 8.3	C/S/I-II	Unclear/37 °C Ringer’s solution 10 mL kg^−1^	21–23 °C/37 °C/drapes	Sitting/L3-4 or L4-5/25 G	C: 0.5% bupivacaine 10 mg + NS
	M_1_: 24 (0)	28.2 ± 7.4					M_1_: 0.5% bupivacaine 10 mg + meperidine 12.5 mg
	M_2_: 24 (0)	27.7 ± 6.4					M_2_: 0.5% bupivacaine 10 mg + meperidine 25 mg
Köroǧlu, *et al*. (2003)^50^	C: 15 (47)	39.2 ± 3.8	Knee arthroscopy/I	Unclear/NS 10 mL kg^−1^	Unclear/unclear/unclear	Sitting/L4-5/22 G	C: 0.5% bupivacaine (H) 10 mg + 0.5 mL NS
	M: 15 (53)	36.3 ± 3.2					M: 0.5% bupivacaine (H) 10 mg + meperidine 25 mg
	F: 15 (60)	35.2 ± 2.5					F: 0.5% bupivacaine (H) 10 mg + fentanyl 25 μg
Murto, *et al*. (1999)^16^	C: 13 (100)	69.2 ± 6.5	TURP/I-III	Diazepam or midazolam/unclear	Unclear/unclear/drapes	Sitting/L2-3 or L3-4/22-27 G	C: 5% lidocaine (H) 75 mg
	M_1_: 14 (100)	68.7 ± 9.4					M_1_: 5% lidocaine (H) 75 mg + meperidine 0.15 mg kg^−1^
	M_2_: 13 (100)	64.2 ± 8.8					M_2_: 5% lidocaine (H) 75 mg + meperidine 0.30 mg kg^−1^
Nag and Gode (1984)^54^	C: 20	Unclear	Below of the umbilicus surgery /unclear	Diazepam/unclear	Unclear/unclear/unclear	Lateral/lumber/22 G	C: 1% bupivacaine (H) 1.2-1.8 mL + 2 mL NS
	M: 20						M: 1% bupivacaine (H) 1.2-1.8 mL + meperidine 6 mg
	Mo: 20						Mo: 1% bupivacaine (H) 1.2-1.8 mL + morphine 1 mg
Rastegarian, *et al*. (2013)^29^	C: 50 (0)	26.3 ± 3.7	C/S/I-II	Unclear/37 °C LR 10 mL kg^−1^	21-23 °C/37 °C/drapes	Sitting/L3-4/25 G	C: 5% lidocaine (H) 75 mg + NS
	M: 50 (0)	27.0 ± 6.1					M: 5% lidocaine (H) 75 mg + meperidine (PF) 0.2 mg kg^−1^
Roy, *et al*. (2004)^57^	C: 20 (0)	32 ± 6.0	C/S/I-II	Unclear/37 °C LR 15 mL kg^−1^	21-23 °C/37 °C/drapes	Sitting/L3-4/27 G	C: 0.75% bupivacaine (H) 10.5 mg + morphine 0.15 mg + NS
	M: 20 (0)	31 ± 5.0					M: 0.75% bupivacaine (H) 10.5 mg + morphine 0.15 mg + meperidine 0.2 mg kg^−1^
Safavi, *et al*. (2014)^30^	C: 40 (72.5)	36 ± 14	Lower limb orthopedic surgery/I-II	NM/37 °C LR 10 mL kg^−1^ h^−1^	21-22 °C/37 °C/Cloth	Sitting/L3-4/22 G	C: 0.5% bupivacaine (H) + NS
	M: 40 (65)	38 ± 15					M: 0.5% bupivacaine (H) + meperidine 0.2 mg kg^−1^
	O: 40 (67.5)	38 ± 17					O: 0.5% bupivacaine (H) + NS + IV ondansetron 8 mg
Safavi, *et al*. (2014)^46^	C: 30 (73.3)	40.1 ± 14.7	Lower limb orthopedic surgery/I-II	NM/37 °C LR 10 mL kg^−1^	21–23°C/37°/blanket	Sitting/L3-4 or L4-5/25 G	C: 0.5% bupivacaine (H) 15 mg + NS
	M: 30 (86.7)	37.2 ± 12.2					M: 0.5% bupivacaine (H) 15 mg + meperidine 0.2 mg kg^−1^
	F: 30 (70)	44.6 ± 16					F: 0.5% bupivacaine (H) 15 mg + fentanyl 20 μg
Shami, *et al*. (2016)^59^	C: 50 (0)	31.8 ± 4.7	C/S/I-II	NM/37 °C LR 500 mL	24–26 °C/unclear/drapes + blanket	Sitting/L3-4 or L4-5/25 G	C: 0.5% bupivacaine (H) 12.5 mg + NS 0.5 mL
	M_1_: 50 (0)	31 ± 5.5					M_1_: 0.5% bupivacaine (H) 12.5 mg + meperidine (PF) 5 mg
	M_2_: 50 (0)	31.5 ± 5.6					M_2_: 0.5% bupivacaine (H) 12.5 mg + meperidine (PF) 10 mg
Tzeng, *et al*. (1987)^33^	C: 20 (0)	39.9	Gynecological surgery/II-III	NM/LR 300 mL	Unclear/unclear/unclear	Lateral/L3-4/24 G	C: tetracaine 10 mg + 2 mL 10% G/W
	M: 20 (0)	39.8					M: tetracaine 10 mg + 2 mL 10% G/W + meperidine 0.25 mg kg^−1^
Wang, *et al*. (2013)^42^	C: 15 (0)	28.6 ± 6.4	C/S/I-II	Unclear/HES 500 mL	24 °C/unclear/unclear	Lateral/L2-3/unclear	C: 0.5% bupivacaine 10 mg
	M_1_: 15 (0)	26.0 ± 4.6					M_1_: 0.5% bupivacaine 10 mg + meperidine 5 mg
	M_2_: 15 (0)	29.1 ± 5.3					M_2_: 0.5% bupivacaine 10 mg+ meperidine 10 mg
	M_3_: 15 (0)	27.6 ± 4.0					M_3_: 0.5% bupivacaine 10 mg + meperidine 15 mg
Yi, *et al*. (2005)^51^	C: 20	64.5 ± 7.7	Unilateral herniorrhaphy/I-II	Midazolam/no	20 ± 2 °C/no/unclear	Lateral/L3-4/25 G	C: 0.5% bupivacaine (H) 13 mg + 0.004 mL kg^−1^ NS
	M: 20	62.9 ± 8.2					M: 0.5% bupivacaine (H) 13 mg + meperidine (PF) 0.2 mg kg^−1^
Yu, *et al*. (2002)^32^	C: 20 (0)	33 ± 6.0	C/S/I-II	Ranitidine/LR 20 mL kg^−1^	Unclear/unclear/unclear	Lateral/L2-3 or L3-4/25 G	C: 0.5% bupivacaine (H) 10 mg + NS
	M: 20 (0)	33 ± 5.0					M: 0.5% bupivacaine (H) 10 mg + meperidine (PF) 10 mg
Zabetian, *et al*. (2013)^48^	C: 35 (0)	27.1 ± 4.2	C/S/I-II	NM/37 °C LR 15 mL kg^−1^	21-23 °C/37 °C/blanket	Sitting/L3-4/25 G	C: 0.5% bupivacaine (H) 10 mg + NS
	M: 35 (0)	28.5 ± 7.3					M: 0.5% bupivacaine (H) 10 mg + meperidine 10 mg

ASA, Physical status classification of American Society of Anesthesiologists; C, control group; C/S, caesarean section; EA, epidural anaesthesia; F, fentanyl group; G, gauge; G/W, glucose water; H, hyperbaric or heavy; HES, hydroxyethyl starch 130/0.4 and sodium chloride injection; HS, Hartmann’s solution; LR, lactated Ringer’s solution; M, meperidine group, Mo, morphine group; NC, no control; NM, no medication; NS, normal saline; OR, operating room; PF, preservative-free; SA, spinal anaesthesia; TURP, trans-urethral resection of prostate; y/o, years old.

.

The quality assessment of RCT methodology is shown in Supplementary Table 1. Acceptable sequence generation was noted in 18 studies^16,25–28,30–32,42–44,46–48,52,55,57,59^, while one had poor quality^29^ and the rest were unclear. How allocation concealment was carried out was clearly described in 11 RCTs^26,28–32,47,48,52,57,59^. Performance bias was found in 5 studies^30,33,50,51,54^, while detection bias was identified in 13 trials^32,33,42–44,49–55,59^. Two studies performed a per-protocol analysis, and 7 patients were withdrawn in total during the follow-up periods^28,45^. Other biases included differences in shivering assessment scales^46,49^, existence of data error^26,47,59^, combination with epidural anaesthesia^28,42,43^, and nondisclosure of intraoperative shivering data^31^ and spinal needle size^28,42,43^.

### Main outcomes with TSA

**Figure 2:**
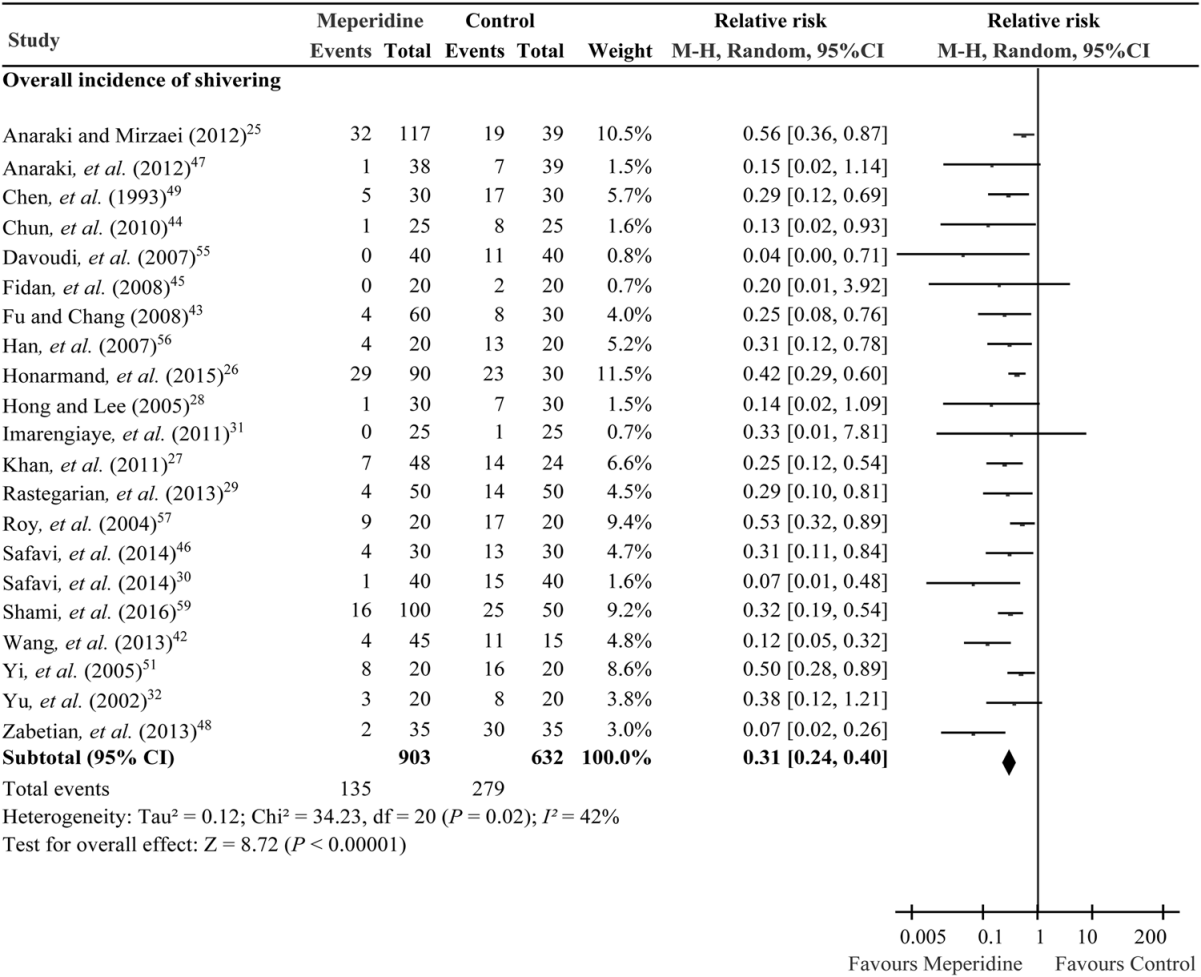
Forest plot comparing adjuvant intrathecal meperidine and control groups on incidence of shivering under spinal anaesthesia.

Trial sequential analysis provides the necessary sample size for our meta-analysis and boundaries that determine whether the evidence in our meta-analysis is reliable and conclusive. The overall incidence of shivering derived from 21 RCTs^25–32,42–49,51,55–57,59^ (n = 1535) was significantly reduced in the adjuvant low dose meperidine groups when compared with control, with an RR of 0.31 (95% CI, 0.24 to 0.40, *P* < 0.00001; *I* ^2^ = 42%) (Fig. 2), relative risk reduction (RRR) of 66.1%, absolute risk reduction (ARR) of 29.2%, and numbers needed to treat (NNTs) 3.4.

**Figure 3:**
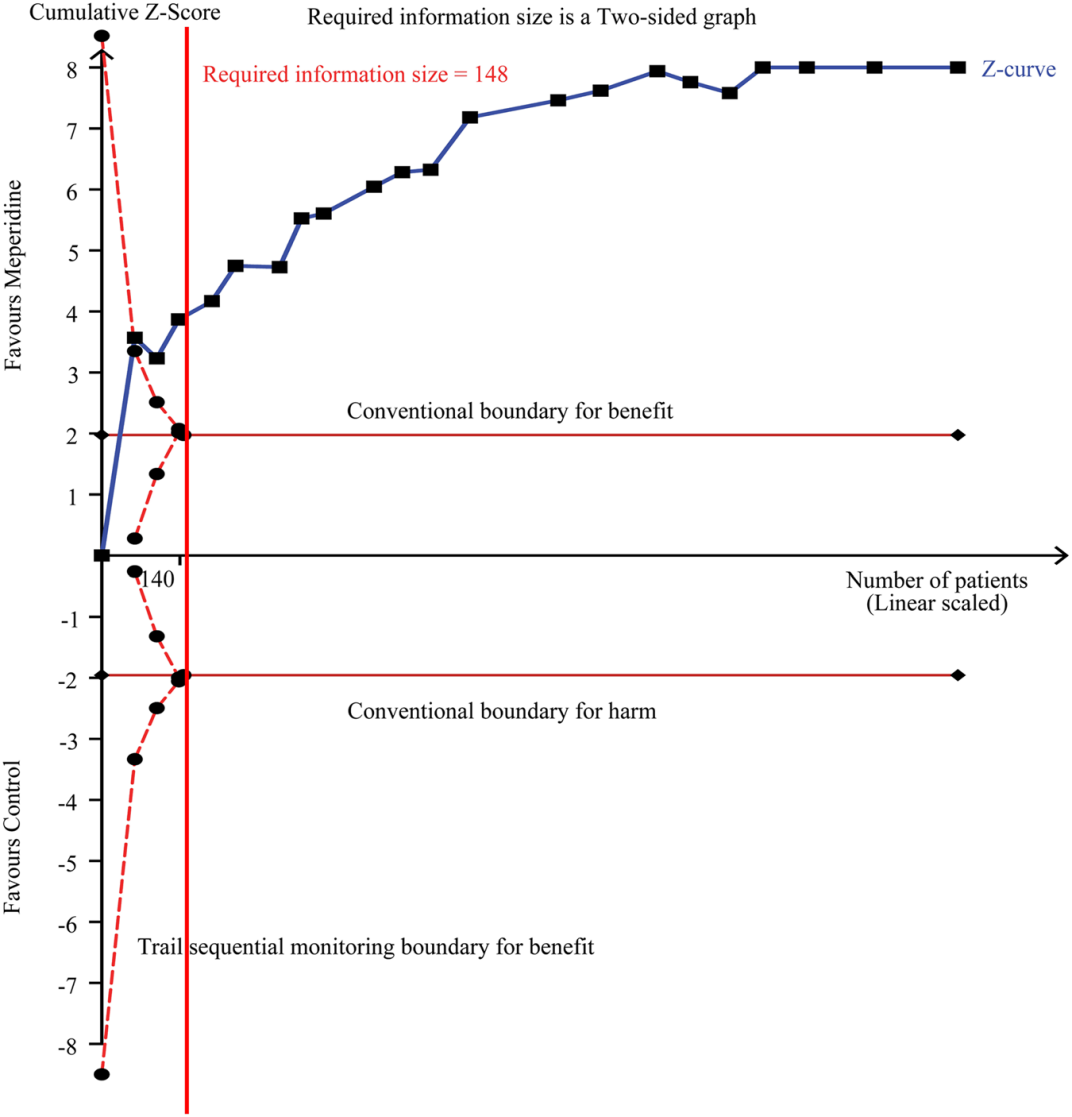
Trial sequential analysis of incidence of shivering under spinal anaesthesia in 21 trials. We calculated an alpha-spending adjusted required information size of 148 patients using α = 0.05 (two-sided), β = 0.20 (power = 80%), diversity (D^2^) = 47%, an anticipated relative risk reduction of 66.1% and an event proportion of 44.2% in the control arm. The cumulative z-curve (blue) was constructed using a random-effects model. If the cumulative z-curve crosses the trial sequential monitoring boundary, a sufficient level of evidence has been reached and no further trials are needed. If the z-curve does not cross the boundary and the required information size has not been reached there is insufficient evidence to reach a conclusion. The required information size (148 patients) was reached and the z-curve crossed the conventional boundary for benefit. The TSA adjusted confidence interval was 0.25 to 0.41.

**Figure 4:**
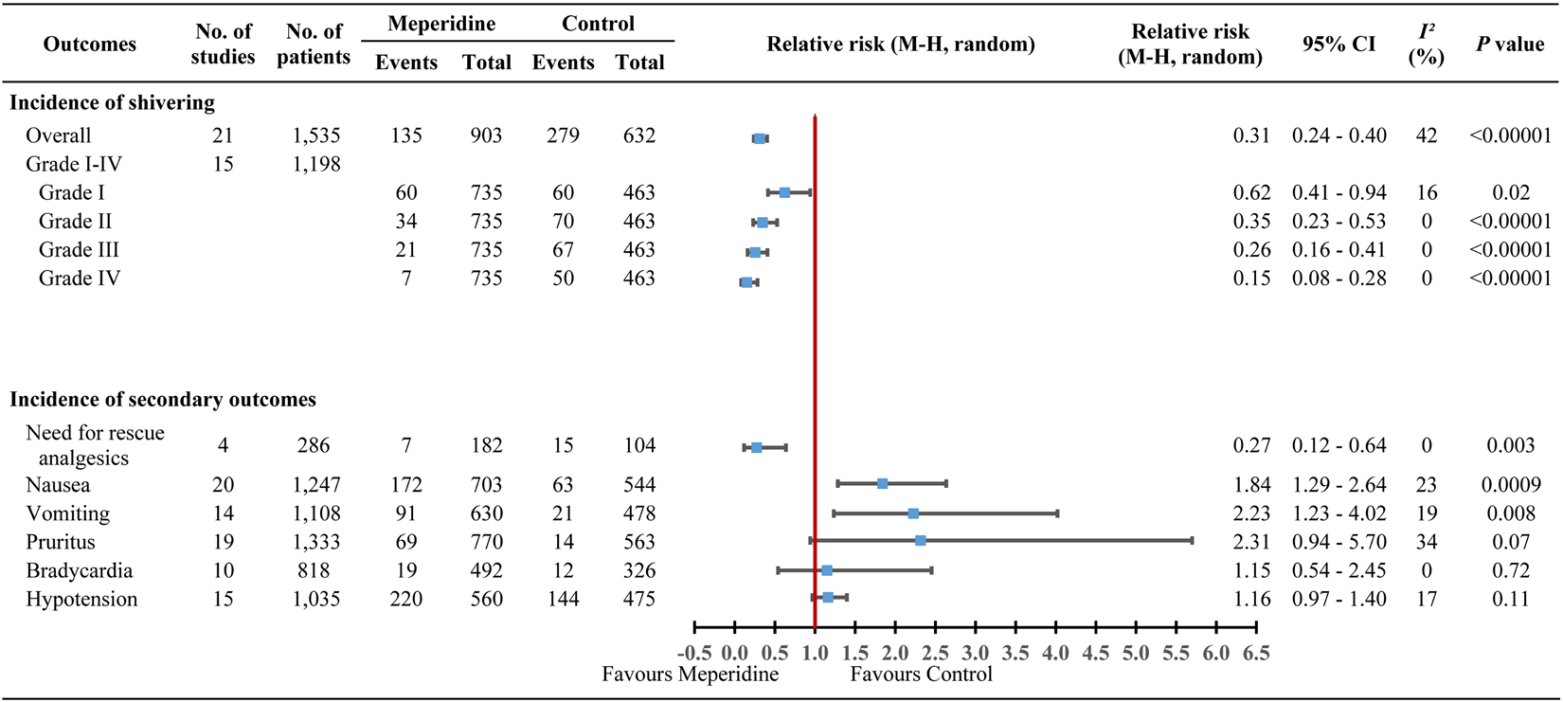
Forest plot comparing adjuvant intrathecal meperidine and control groups indicating intensity of shivering graded I-IV and secondary outcomes.

If the cumulative z-curve crosses the trial sequential monitoring boundary, a sufficient level of evidence has been reached and no further trials are needed. If the z-curve does not cross the boundary and the required information size has not been reached there is insufficient evidence to reach a conclusion. TSA showed the required information size of 148 patients was reached and the cumulative z-curve was touched and crossed the trial sequential monitoring boundary for benefits. The TSA adjusted RR was 0.32 (95% CI, 0.25 to 0.41; 1535 patients, 21 trials) (Fig. 3 and Table 2), providing firm evidence for shivering prevention. Moreover, 15 trials^25–30,42,43,46,48,49,51,56,57,59^ (n = 1198) have further evaluated the preventive effect of adjuvant low dose intrathecal meperidine to the incidence of shivering based on a 4-level intensity scale^60^, showing that the RRs were 0.62 (95% CI, 0.41 to 0.94, *P = *0.02) for grade I, 0.35 (95% CI, 0.23 to 0.53, *P* < 0.00001) for grade II, 0.26 (95% CI, 0.16 to 0.41, *P* < 0.00001) for grade III, and 0.15 (95% CI, 0.08 to 0.28, *P* < 0.00001) for grade IV shivering (Fig. 4).

**Table 2:** Trial sequential analysis of incidence of outcomes under spinal anaesthesia.

Outcomes	No. of studies	Actual sample size	Event proportion in intervention arm (%)	Event proportion in control arm (%)	D^2^ (%)	RIS (sample size)	Z-curve crosses the conventional boundary^b^	TSA adjusted RR	TSA adjusted 95% CI	*P* value
Shivering	21	1,535	14.8	43.6	47	148	Yes	0.32	0.25–0.41	<0.0001
Need for rescue analgesics	4	286	3.8	14.4	0	232	Yes	0.27	0.08–0.91	0.0027
Nausea	20	1,247	24.45	11.58	29	396	Yes	1.72	1.33–2.23	<0.0001
Vomiting	14	1,108	14.44	4.39	0	266	Yes	1.96	1.20–3.21	0.0047
Pruritus	19	1,333	8.96	2.49	0	405	No	1.42	0.87–2.34	0.1443
Bradycardia^b^	10	818	3.86	3.68	0	351,540	No	NA	NA	0.8048
Hypotension	15	1,035	39.29	30.32	28	1,228	No	1.15	0.95–1.40	0.1002

TSA calculated an alpha-spending adjusted required information size using α = 0.05 (two-sided), β = 0.20 (power = 80%) and D2, the cumulative z-curve was constructed using a random-effects model.

^a^The cumulative z-score reaches significance by crossing both the conventional boundaries.

^b^TSA with alpha-spending adjusted confidence interval cannot be calculated, boundary required sample size is ignored due to too little information (0.23%).

CI, confidence interval; D2, diversity; NA, not applicable; TSA, trial sequential analysis; RIS, required information size; RR, relative risk; z-curve, cumulative z-curve.

The need for intraoperative rescue analgesics was reduced in the adjuvant low dose meperidine groups with RR 0.27 (95% CI, 0.12 to 0.64, *P = *0.003), RRR 73.3%, ARR 10.6%, and NNTs 9.5 (Fig. 4). Our result has reached the required information size (n = 232) of TSA, and the z-curve has also crossed the conventional boundary of benefit with an adjusted RR, 0.27 (95% CI, 0.08 to 0.91; 286 patients, 4 trials) (Table 2).

Adjuvant low dose intrathecal meperidine also increased the risk of nausea with RRs of 1.84 (95% CI, 1.29 to 2.64, *P = *0.0009; *I* ^2^ = 23%; number needed to harm [NNH]: 7.8), and in vomiting with RRs of 2.23 (95% CI, 1.23 to 4.02, *P = *0.008; *I* ^2^ = 19%; NNH: 9.9) (Fig. 4). Their TSA required information size (396 and 266 patients) were both reached and their z-curve also crossed the conventional boundary of harm, with a TSA adjusted RR, 1.72 (95% CI, 1.33 to 2.23; 1247 patients, 20 trials) on nausea and 1.96 (95% CI, 1.20 to 3.21; 1108 patients, 14 trials) on vomiting, respectively (Table 2). These results provided a sufficient level of evidence implying that intrathecal meperidine may increase the risks of nausea and vomiting.

The RR of the incidence of pruritus when using adjuvant low dose intrathecal meperidine was 2.31 (95% CI, 0.94 to 5.70, *P = *0.07; *I* ^2^ = 34%; NNH: 15.4) (Fig. 4) and the TSA required information size of 405 patients was reached. Such a conclusion was echoed by the z-curve without crossing the conventional boundary of harm (TSA adjusted RR, 1.42, 95% CI: 0.87 to 2.34; 1333 patients, 19 trials) (Table 2). This result provided firm evidence showing that intrathecal meperidine may not increase bring forth the harm of pruritus. Two drug-related morbidities when using adjuvant low dose intrathecal meperidine, bradycardia and hypotension, were not increased in comparison with control groups; the RRs for these were 1.15 (95% CI, 0.54 to 2.45, *P = *0.72; *I* ^2^ = 0%; NNH: 553.1) and 1.16 (95% CI, 0.97 to 1.40, *P = *0.11; *I* ^2^ = 17%; NNH: 11.1), respectively. However, their TSA required information size were not reached and their z-curves did not cross the conventional boundary: TSA adjusted 95% CI cannot be calculated due to too little information on bradycardia (actual vs. required information patients size = 818/351,540 = 0.23%; 10 trials), and TSA adjusted RR, 1.15 (95% CI, 0.95 to 1.40; 1035 patients, 15 trials) on hypotension (Fig. 4 and Table 2). None of the boundaries for benefit or harm was crossed, showing insufficient evidence to allow us to conclude whether the intervention was harmful on the above two results.

### Publication bias

**Figure 5:**
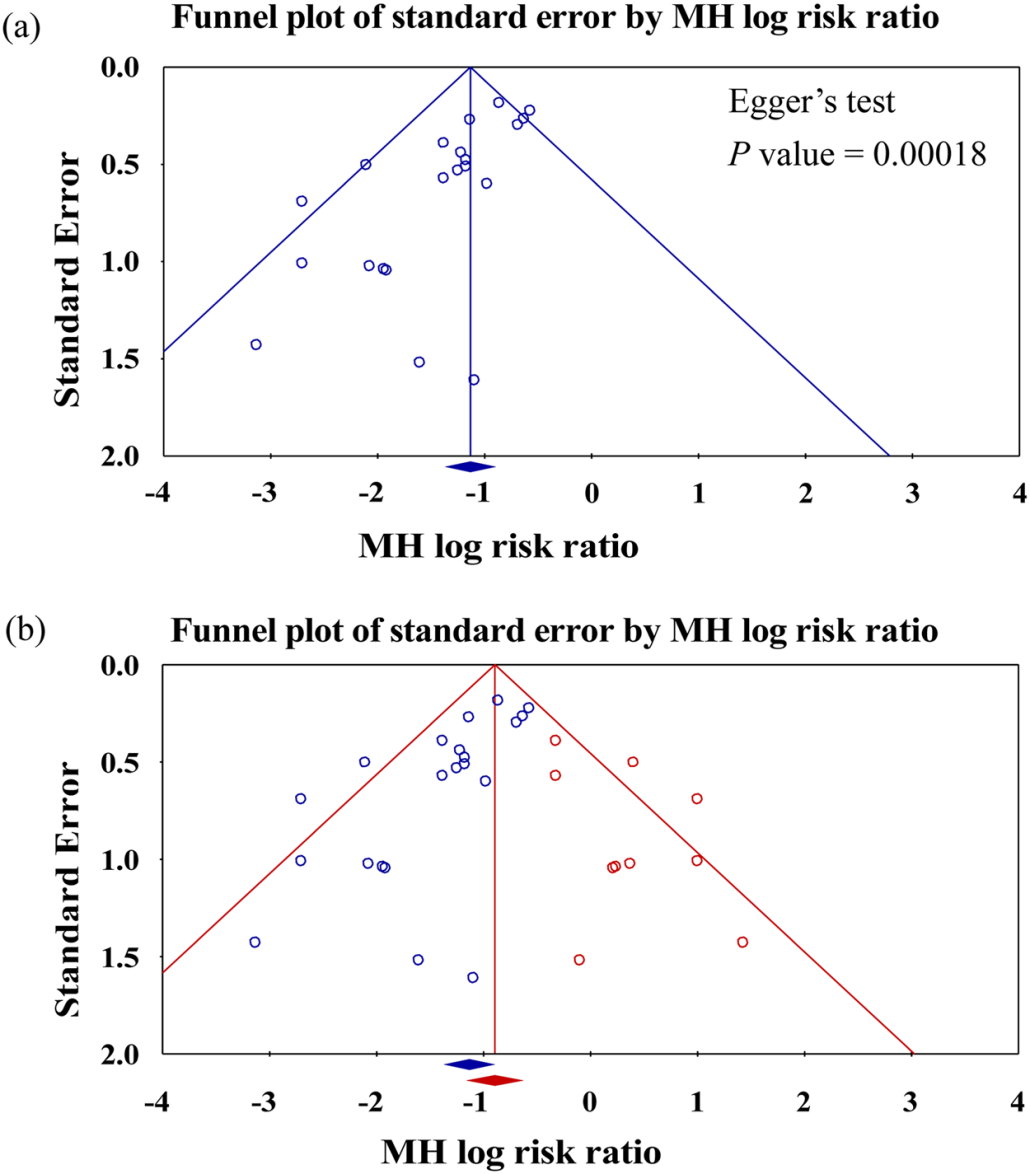
Funnel plots was applied to assess publication bias which were plotted in the log risk ratios against their standard errors and estimating the number of missing studies that might exist in a meta-analysis and the effect that these studies might have had on its outcome. (**a**) Funnel plot with 95% confidence limits for testing publication bias; (**b**) Funnel plot of all studies with 95% CI, including hypothetical studies using ‘trim and fill’ method (in red) for adjusting publication bias. After adjusting for missing studies, we noted that the point estimate of the overall effect size is approximately correct and coverage of the effect size confidence intervals is substantially improved. The results showed that publication bias or another confounding variable should be considered, but would not be a major influencing factor for the intervention effect. That is, the publication bias did not affect our major outcomes.

The funnel plot showed the asymmetric distribution of studies and Egger’s test was significant (*P = *0.00018; Fig. 5a). The “trim and fill” method results showed ten necessary studies were missed. After filling these ten with comprehensive analysis, the funnel plot showed improved symmetry (Fig. 5b). Under the random effects model, the Mantel-Haenszel (MH) log risk ratio and 95% confidence interval for the combined studies is 0.32 (0.25 to 0.41). Imputed MH log risk ratio by “trim and fill” method is 0.41 (0.31 to 0.53). The results showed that publication bias or another confounding variable should be considered, but would not be a major influencing factor for the intervention effect. That is, the publication bias did not affect our major outcomes.

### Subgroup analyses

Subgroups of different surgical types and intrathecal meperidine doses were analysed (Table 3). The anti-shivering effect of intrathecal meperidine was observed in all four types of surgery, with RRs 0.30 (95% CI, 0.21 to 0.43, *P* < 0.00001) in caesarean section, 0.30 (95% CI, 0.14 to 0.63, *P* = 0.002) in lower limb orthopaedic surgery, 0.11 (95% CI, 0.03 to 0.38, *P* = 0.0006) in urology, and 0.42 (95% CI, 0.25 to 0.70, *P* = 0.0008) in other miscellaneous surgical patients, respectively. The benefit of reducing the need of rescue analgesics and the morbidities of nausea and vomiting were only noted in the subgroup of caesarean section. The subgroup analysis of intervention dose indicated that dose equal to or less than 0.2 mg kg^−1^ or 12.5 mg (Group I) could provide similar anti-shivering and analgesic effect (reduced need for rescue analgesics) as dose more than 0.2 mg kg^−1^ or 12.5 mg, up to 0.5 mg kg^−1^ or 25 mg (Group II) in comparison with the control (Table 3). However, it did not decrease the occurrence of drug-related adverse events as anticipated (Supplementary Figure 1

**Table 3:** Subgroup analyses: the effect of surgery type and different dose levels of adjuvant intrathecal meperidine. ^a^Includes transurethral resection of the prostate and suprapubic prostatectomy; ^b^Includes surgery of lower limbs or abdomen and herniorrhaphy; Group I, adjuvant intrathecal meperidine ≦ 0.2 mg kg^−1^ or ≦ 12.5 mg; Group II, adjuvant intrathecal meperdine >0.2 mg kg^−1^ or >12.5 mg; NA, not applicable; RR, relative risk.

Subgroup	No of studies	Overall incidence of shivering				Incidence of need for rescue analgesics				Incidence of nausea				Incidence of vomiting			
		RR	95% CI	*I* ^2^	*P*	RR	95% CI	*I* ^2^	*P*	RR	95% CI	*I* ^2^	*P*	RR	95% CI	*I* ^2^	*P*
**Overall**	28	0.31	0.24–0.40	42%	<0.00001	0.27	0.12–0.64	0%	0.003	1.84	1.29–2.64	23%	0.0009	2.23	1.23–4.02	19%	0.008
**Surgery type**																	
Caesarean section^25,27–29,31,32,42,43,48,52,53,56–59^	15	0.3	0.21–0.43	49%	<0.00001	0.26	0.10–0.66	0%	0.005	1.93	1.17–3.16	46%	0.009	2.6	1.16–5.85	44%	0.02
Orthopedic surgery^26,30,45,46,50^	5	0.3	0.14–0.63	43%	0.002	0.33	0.04–2.94	NA	0.32	3.65	0.58–23.05	34%	0.17	NA	NA	NA	NA
^a^Urology surgery^16,44,47,55^	4	0.11	0.03–0.38	0%	0.0006	0.41	0.17–0.98	NA	0.05	3.5	0.19–63.16	NA	0.4	2	0.19–21.18	NA	0.56
^b^Other surgery^33,49,51,54^	4	0.42	0.25–0.70	9%	0.0008	NA	NA	NA	NA	1.82	0.83–4.00	0%	0.14	1.96	0.36–10.71	0%	0.44
**Meperidine dose**																	
Group I^16,25–32,42–46,48,49,51,52,54,56,57,59^	22	0.34	0.25–0.46	47%	<0.00001	0.28	0.09–0.86	0%	0.03	1.73	1.26–2.38	0%	0.0008	2.58	1.34–4.98	0%	0.005
Group II^16,25–27,33,42,47,50,53,55,58^	11	0.17	0.08–0.38	0%	<0.0001	0.26	0.07–0.94	NA	0.04	1.95	1.07–3.56	40%	0.03	1.98	0.72–5.44	42%	0.19

).

### Meta-regression analyses

In the univariate meta-regression analyses, age, meperidine dose, and sample size of each study were not influenced by the heterogeneity of sources, whose coefficients were 0.002 (*P* = 0.94), 2.68 (*P* = 0.42), and 0.007 (*P* = 0.33), respectively (Supplementary Figure 2). These three variables evaluated were all not significantly associated with shivering prevention.

### Sensitivity analyses

The sensitivity analysis of the potential bias is shown in Table 4

**Table 4:** Sensitivity Analyses: The effect of potential biases on primary outcomes of adjuvant intrathecal meperidine. ^a^Excluded high or unclear risk; ^b^Excluded with unclear information; ASA, Physical status classification of American Society of Anesthesiologists; CI, confidence interval; G, gauge; LA, local anaesthetics; N/A, not applicable; RR, relative risk; y/o, years old.

Potential bias or limitations excluded	No. of studies	Overall incidence of shivering				Incidence of need for rescue analgesics				Incidence of nausea				Incidence of vomiting			
		RR	95% CI	*I* ^2^	*P*	RR	95% CI	*I* ^2^	*P*	RR	95% CI	*I* ^2^	*P*	RR	95% CI	*I* ^2^	*P*
**Overall**	28	0.31	0.24–0.40	42%	<0.00001	0.27	0.12–0.64	0%	0.003	1.84	1.29–2.64	23%	0.0009	2.23	1.23–4.02	19%	0.008
**RCT quality** ^**a**^																	
Selection bias^16,25,27,29,33,42–46,49–51,53–56,58^	18	0.28	0.20–0.40	54%	<0.00001	0.26	0.10–0.66	0%	0.005	2.15	1.27–3.63	24%	0.004	2.91	1.60–5.28	0%	0.0005
Performance bias^30,33,50,51,54^	23	0.31	0.23–0.40	37%	<0.00001	0.27	0.12–0.64	0%	0.003	2.1	1.30–3.38	41%	0.002	2.26	1.17–4.38	27%	0.02
Detection bias^32,33,42–44,49–55,59^	15	0.32	0.22–0.46	48%	<0.00001	0.27	0.11–0.65	0%	0.004	4.18	1.06–16.55	69%	0.04	3.32	1.04–10.66	54%	0.04
Attrition bias^28,45^	26	0.31	0.24–0.41	45%	<0.00001	0.26	0.10–0.66	0%	0.005	1.71	1.22–2.39	18%	0.002	2.23	1.23–4.02	19%	0.008
Other bias^26,28,31,42,43,46,47,49,59^	19	0.3	0.19–0.47	58%	<0.00001	0.3	0.12–0.72	0%	0.008	2.45	1.39–4.34	44%	0.002	2.65	1.04–6.72	42%	0.03
**Participants** ^**b**^																	
ASA III^16,33,44,45,47,54,55^	21	0.33	0.25–0.42	43%	<0.00001	0.26	0.10–0.66	0%	0.005	1.85	1.22–2.81	32%	0.004	2.38	1.13–4.98	37%	0.02
Age ≥ 65 y/o^16,26,44,47,51,52,54,55^	20	0.29	0.21–0.40	41%	<0.00001	0.27	0.12–0.64	0%	0.003	2.04	1.29–3.23	40%	0.002	2.33	1.23–4.52	28%	0.01
**Technique** ^**b**^																	
No bupivacaine^16,29,33,47,49,55,58^	21	0.32	0.24–0.43	46%	<0.00001	0.27	0.12–0.64	0%	0.003	1.94	1.35–2.77	8%	0.0003	2.98	1.61–5.52	0%	0.0005
LA contain other drugs^52,57,58^	25	0.29	0.22–0.39	39%	<0.00001	0.27	0.12–0.64	0%	0.003	1.84	1.37–2.47	0%	<0.0001	2.93	1.69–5.09	0%	0.0001
Needle size ≤ 24 G^16,28,30,33,42,43,49,50,52,54^	18	0.36	0.27–0.46	35%	<0.00001	0.21	0.07–0.67	25%	0.008	2.38	1.28–4.44	58%	0.006	2.44	1.19–5.01	35%	0.01
No prehydration^16,45,51,54^	24	0.29	0.22–0.39	45%	<0.00001	0.16	0.03–0.97	51%	0.05	1.85	1.23–2.79	32%	0.003	2.23	1.23–4.02	19%	0.008
No drapes^26,31–33,42,43,45,47,49–55,58^	13	0.3	0.21–0.44	50%	<0.00001	0.29	0.11–0.77	0%	0.01	3.75	1.37–10.21	59%	0.01	3.05	1.65–5.64	0%	0.0004
No warm prehydretion^16,28,31–33,42–45,47,49–54,56,58^	10	0.32	0.22–0.47	62%	<0.00001	0.29	0.10–0.80	NA	0.02	4.87	0.9–26.34	70%	0.07	2.97	1.64–5.38	0%	0.003

. After considering 13 items of potential bias in three categories, all results remained consistently significant except the incidence of nausea, which showed no statistical significance when excluding trials without using warm pre-hydration.

## Discussion

We demonstrated that low dose intrathecal meperidine as an adjuvant for spinal anaesthesia could effectively prevent shivering and reduce need for rescue analgesics, yet still might increase risk of nausea and vomiting. TSA further provided a sufficient level of evidence with power of accuracy and reliability for the meta-analysis^37,61^.

Previous studies noted the anti-shivering effect of intrathecal opioids^62^. Various opioids were studied by Pöpping. *et al*. without subgroup analysis, and meperidine was recruited in only two trials^62^. Furthermore, since the dose of local anaesthetics in experimental groups was deliberately decreased, whether the reduced risks (e.g., shivering and nausea) were related to the adjuvant opioid or reduced local anaesthetic was unclear^62^. Thus comparing meperidine as adjuvant under an equal amount of intrathecal local anaesthetics guided our meta-analysis design. Feng. *et al*. investigated primarily sulfentanil and showed no beneficial effect while also revealing increased the incidence of pruritus^63^. Shortcomings of that study included mixed use of spinal and epidural anaesthesia and mixed local anaesthetic drugs which have been carefully avoided in our sensitivity analyses^63^. Therefore, our results on intrathecal meperidine for the prevention on shivering under spinal anaesthesia would be more reliable due to meticulous management of this bias.

The mechanism of shivering during spinal anaesthesia is multifactorial. Sympathetic blockade caused by spinal anaesthesia impairs compensatory vasoconstriction and autonomic regulation below the level of the blockade^1,8^, and blunts thermoregulatory processing^2,3^ leading to vasodilation, heat loss and hypothermia: all these factors might contribute to shivering. Meperidine is the most common intravenousdrug used for treating and preventing shivering, as its equi-analgesic dose is much more efficient than other opioids such as fentanyl, alfentanil, sufentanil or morphine in preventing shivering^15,64,65^. Meperidine is the only opioid that is an agonist at both the μ and κ receptors closely related to the pathogenesis of shivering by reducing the shivering threshold and triggering decreasedcore temperature, constitutes its anti-shivering effect^15,65–67^.

Nausea and vomiting are common opioid-related side effects, but their mechanisms are extremely complex^68–71^. The increased incidence of nausea and vomiting associated with intrathecal meperidine may relate to several issues. First, intrathecal meperidine could be deemed as an analgesic independently, so a higher level of anaesthesia might cause systemic hypotension, nausea and vomiting when adjuvant was given. However, none of our recruited studies had significant differences regarding anaesthetic level between the control and the meperidine group. Future studies could focus on the optimal dose of local anaesthetics and intrathecal meperidine and possible interactions. Furthermore, like morphine and other opioids, intrathecal meperidine also showed its central effect inducing nausea and vomiting^13,32,72^.

Subgroup analyses demonstrated that the protective effect of adjuvant low dose intrathecal meperidine against shivering was demonstrated among caesarean section, urological, other lower abdominal and lower limb orthopaedic surgeries. It is particularly beneficial to elderly and obstetric patients who are vulnerable to shivering-induced oxygen consumption, metabolic demands and cardiovascular morbidities^10,73–76^. The sensitivity analyses illustrated that both therapeutic and adverse effects of intrathecal meperidine are unaffected by anaesthetic techniques, perioperative care, and patient age or physical status. The benefit of reduction of rescue analgesic in low dose intrathecal meperidine with less nausea and vomiting could not be generalized to all surgeries due to the limited RCTs included. Furthermore, reducing meperidine dosages could not prevent nausea and vomiting as anticipated. Thi. *et al*. showed that the dose-dependent analgesic effect of intrathecal meperidine lower than 0.5 mg kg^−1^ would not aggravate nausea and vomiting^24^. Although we demonstrated that dose equal to or less than 0.2 mg kg^−1^ or 12.5 mg could provide anti-shivering and adequate analgesia as effective as dose more than 0.2 mg kg^−1^ or 12.5 mg, an optimal dose of low dose intrathecal meperidine was not yet identified. Since the benefits of shivering protection and rescue analgesics reduction were not overwhelmed by nausea and vomiting, the potential utility of such interventions could be considered in clinical practice.

The are several strengths to this review. Using Cochrane methodology for a non-restricted up-to-date literature search without limitation by language or country of publication (8 countries and 5 languages) is one of our major strengths. We also attempted to correct publication bias using the’trim and fill’ method^40,41^ reducing possible influence from selection reporting bias. We analysed the incidence of shivering as well as the effect on different grades of shivering, conducted sensitivity analyses to improve the reliability of our results, and performed TSA to reduce systematic and random errors and identify the threshold of statistical significance^37,61^. TSA was applied to account for scattered data and repetitive testing on accumulated data, to calculate the required information size, and to confirm the cumulative z-curve crossing the boundary of benefit or harm in the listed outcomes^37,61^. This meta-analysis with TSA provided us robust results and indicted that no further trials about adjuvant intrathecal meperidine on shivering prevention are needed in the current future. This is the first systematic review and meta-analysis using TSA to validate the benefits and harms of intrathecal meperidine for patients undergoing spinal anaesthesia.

Our study has several limitations. First, although various biases among all RCTs included have been considered under sensitivity analysis and three possible covariables were also assessed by meta-regression, results remained moderately heterogenious and other covaribles were unidentified. This implies that the conclusion needs to be carefully intepreted and applied in clinical practice due to the existence of qualitative and statistical heterogeneity for our overall incidence of shivering as well as specific findings such as pruritus, nausea and vomiting. Second, as this study specified single medication through single route,the dose-response effect of adjuvant intrathecal meperidine with local anaesthetics remains unclear and needs further investigation using network meta-analysis^77–79^. Third, local anaesthetics *per se* used in spinal anaesthesia could induce sympathetic blockade, hypotension and nausea/vomiting, which might interfere with observations regarding side effects induced by intrathecal meperidine. Fourth, although subgroup analysis for types of surgeries has been considered, caesearean section surgeries contributed the majority of the database. The application of the study outcome to other surgeries needs to be further evaluated.

## Conclusion

Our meta-analysis with TSA validates the effectiveness of shivering prevention by adjuvant low dose intrathecal meperidine with local anaesthetics for spinal anaesthesia with reduced intraoperative need for rescue analgesics, but it also noted increased incidence of nausea and vomiting. Further larger RCTs or network meta-analyses are needed to evaluate different doses of intrathecal meperidine as well as other routes of administration to help anaesthesiologists provide better care in clinical practice.

## Electronic supplementary material

Supplementary Information
