# Supplementary material for: Preventing shivering with adjuvant low dose intrathecal meperidine: A meta-analysis of randomized controlled trials with trial sequential analysis
Source: Sci Rep. 2017 Nov 10;7:15323. doi: 10.1038/s41598-017-14917-5 (PMC5681692; doi:10.1038/s41598-017-14917-5)
Supplement: Supplementary file 1 — Supplementary Information [file 41598_2017_14917_MOESM1_ESM.pdf]

Preventing shivering with adjuvant low dose intrathecal meperidine: A meta-analysis of randomized controlled trials with trial sequential analysis.

Yu-Cih Lin, RNA[1,2]; Chien-Yu Chen, MD, PhD[1,3,4]; Yuan-Mei Liao, RN, PhD[2,5]; Alan Hsi-Wen Liao, MBchB[1]; Pi-Chu Lin, RN, EdD[2,6]; Chuen-Chau Chang, MD, PhD[1,2,3,*]

[1]Department of Anaesthesiology, Taipei Medical University Hospital, Taipei, 110, Taiwan
[2]School of Nursing, College of Nursing, Taipei Medical University, Taipei, 110, Taiwan
[3]Department of Anaesthesiology, School of Medicine, College of Medicine, Taipei Medical University, Taipei, 110, Taiwan
[4]Graduate Institute of Humanities in Medicine, Taipei Medical University, Taipei, 110, Taiwan
[5]Institute of Clinical Nursing, School of Nursing, National Yang-Ming University, Taipei City, 112, Taiwan
[6]Master Program in Long-Term Care, College of Nursing, Taipei Medical University, Taipei, 110, Taiwan

Corresponding author:
Chuen-Chau Chang,
Taipei Medical University Hospital
252 Wuxing St., Taipei 11031, Taiwan
Tel.: 886-2-2737-2181, ext. 8310
Fax: 886-2-2736-7344
E-mail: nekota@tmu.edu.tw (Chuen-Chau Chang)

Short title: Preventing shivering with adjuvant intrathecal meperidine
Word count for abstract: 208
Word count for text: 3815 (including Methods)

Supplementary Table 1. Risk of bias of selection randomized controlled trials.

Study (year)	Random sequence generation (selection bias)	Allocation concealment (selection bias)	Blinding of participants and personnel (performance bias)	Blinding of outcome assessment (detection bias)	Incomplete outcome data (attrition bias)	Selective reporting (reporting bias)	Other bias
Anaraki and Mirzaei (2012)[25]	L	U	L	L	L	L	L
Anaraki, *et al.* (2012)[47]	L	L	L	L	L	L	H[a]
Chen, *et al.* (1993)[49]	U	U	L	U	L	L	H[b]
Choi, *et al.* (2000)[53]	U	U	L	U	L	L	L
Chun, *et al.* (2010)[44]	L	U	L	U	L	L	L
Chung, *et al.* (1997)[52]	L	L	L	U	L	L	L
Davoudi, *et al.* (2007)[55]	L	U	L	U	L	L	L
Farzi, *et al.* (2014)[58]	U	U	L	L	L	L	L
Fidan, *et al.* (2008)[45]	U	U	L	L	H	L	L
Fu and Chang (2008)[43]	L	U	L	U	L	L	H[d, g]
Han, *et al.* (2007)[56]	U	U	L	L	L	L	L
Honarmand, *et al.* (2015)[26]	L	L	L	L	L	L	H[c]
Hong and Lee (2005)[28]	L	L	L	L	H	L	H[d, g]
Imarengiaye, *et al.* (2011)[31]	L	L	L	L	L	L	H[e]
Khan, *et al.* (2011)[27]	L	U	L	L	L	L	L
Köroğlu, *et al.* (2003)[50]	U	U	U	U	L	L	L
Murto, *et al.* (1999)[16]	L	U	L	L	L	L	L
Nag and Gode (1984)[54]	U	U	U	U	L	L	L
Rastegarian, *et al.* (2013)[29]	H	L	L	L	L	L	L
Roy, *et al.* (2004)[57]	L	L	L	L	L	L	L
Safavi, *et al.* (2014)[30]	L	L	U	L	L	L	L
Safavi, *et al.* (2014)[46]	L	U	L	L	L	L	U[f]
Shami, *et al.* (2016)[59]	L	L	L	U	L	L	H[a]
Tzeng, *et al.* (1987)[33]	U	U	U	U	L	L	L
Wang, *et al.* (2013)[42]	L	U	L	U	L	L	U[d, g]
Yi, *et al.* (2005)[51]	U	U	U	U	L	L	L
Yu, *et al.* (2002)[32]	L	L	L	U	L	L	L
Zabetian, *et al.* (2013)[48]	L	L	L	L	L	L	L

H, high risk; L, low risk; U, unclear risk; [a] Data error noted in manuscript; [b] Different shivering assessment scale; [c] Data errors noted on table 1; [d] Spinal and epidural anaesthesia combined; [e] Only post-operation shivering assessed; [f] No indication of what kind of shivering assessment scale adopted; [g] Spinal needle size unclear

Subgroup	Group I Events	Group I Total	Group II Events	Group II Total	Weight	Relative risk M-H,Random,95%CI
**1.Incidence of shivering**						
Anaraki and Mirzaei (2012)[25]	15	39	17	78	41.5%	1.76 [0.99, 3.15]
Honarmand, *et al.* (2015)[26]	26	60	3	30	27.4%	4.33 [1.43, 13.17]
Khan, *et al.* (2011)[27]	2	24	5	24	19.2%	0.40 [0.09, 1.86]
Wang, *et al.* (2013)[42]	3	30	1	15	11.9%	1.50 [0.17, 13.23]
**Subtotal (95% CI)**		**153**		**147**	**100.0%**	**1.66 [0.71, 3.91]**
Total events	46		26			
Heterogeneity: Tau² = 0.37; Chi² = 6.17, df = 3 (*P* = 0.10); I^2 = 51%						
Test for overall effect: Z = 1.17 (*P* = 0.24)						
**2. Incidence of need for analgesics**						
Anaraki and Mirzaei (2012)[25]	2	39	4	78	43.7%	1.00 [0.19, 5.22]
Murto, *et al.* (1999)[16]	5	14	2	13	56.3%	2.32 [0.54, 9.95]
**Subtotal (95% CI)**		**53**		**91**	**100.0%**	**1.61 [0.54, 4.79]**
Total events	7		6			
Heterogeneity: Tau² = 0.00; Chi² = 0.56, df = 1 (*P* = 0.45); I^2= 0%						
Test for overall effect: Z = 0.85 (*P* = 0.39)						
**3. Incidence of nausea**						
Khan, *et al.* (2011)[27]	18	24	5	24	38.4%	3.60 [1.60, 8.12]
Murto, *et al.* (1999)[16]	2	13	1	14	24.4%	2.15 [0.22, 21.03]
Wang, *et al.* (2013)[42]	5	30	7	15	37.1%	0.36 [0.14, 0.94]
**Subtotal (95% CI)**		**67**		**53**	**100.0%**	**1.35 [0.24, 7.69]**
Total events	25		13			
Heterogeneity: Tau² = 1.89; Chi² = 13.05, df = 2 (*P* = 0.001); I^2 = 85%						
Test for overall effect: Z = 0.33 (*P* = 0.74)						
**4. Incidence of vomiting**						
Anaraki and Mirzaei (2012)[25]	6	39	24	78	54.0%	0.50 [0.22, 1.12]
Khan, *et al.* (2011)[27]	11	24	1	24	46.0%	11.00 [1.54, 78.67]
**Subtotal (95% CI)**		**63**		**102**	**100.0%**	**2.08 [0.09, 49.90]**
Total events	17		25			
Heterogeneity: Tau² = 4.71; Chi² = 9.00, df = 1 (*P* = 0.003); I^2 = 89%						
Test for overall effect: Z = 0.45 (*P* = 0.65)						
**5. Incidence of pruritus**						
Anaraki and Mirzaei (2012)[25]	11	39	34	78	100.0%	0.65 [0.37, 1.13]
Khan, *et al.* (2011)[27]	0	24	0	24		Not estimable
Murto, *et al.* (1999)[16]	0	13	0	14		Not estimable
**Subtotal (95% CI)**		**76**		**116**	**100.0%**	**0.65 [0.37,1.13]**
Total events	11		34			
Heterogeneity: Not applicable						
Test for overall effect: Z = 1.52 (*P* = 0.13)						
**6.Incidence of bradycardia**						
Anaraki and Mirzaei (2012)[25]	0	39	0	78		Not estimable
Khan, *et al.* (2011)[27]	1	24	0	24	28.2%	3.00 [0.13, 70.16]
Murto, *et al.* (1999)[16]	6	13	1	14	71.8%	6.46 [0.89, 46.70]
**Subtotal (95% CI)**		**76**		**116**	**100.0%**	**5.20 [0.97, 27.79]**
Total events	7		1			
Heterogeneity: Tau² = 0.00; Chi² = 0.16, df = 1 (*P* = 0.69); I^2= 0%						
Test for overall effect: Z = 1.93 (*P* = 0.05)						
**7. Incidence of hypotension**						
Anaraki and Mirzaei (2012)[25]	0	39	0	78		Not estimable
Khan, *et al.* (2011)[27]	11	24	12	24	100%	0.92 [0.51, 1.66]
**Subtotal (95% CI)**		**63**		**102**	**100%**	**0.92 [0.51, 1.66]**
Total events	11		12			
Heterogeneity: Not applicable						
Test for overall effect: Z = 0.29 (*P* = 0.77)						

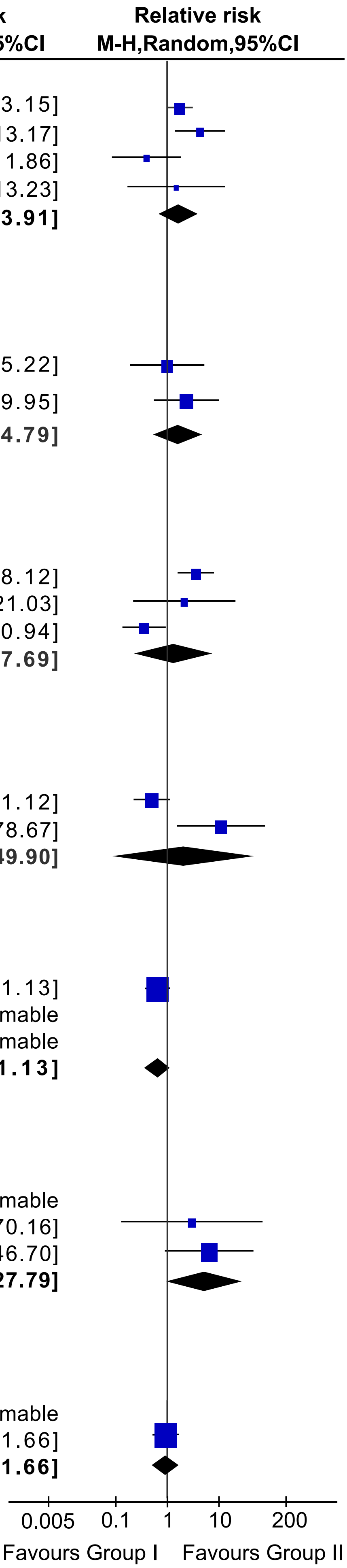

Supplementary Figure 1. Comparing dose equal to or less than 0.2 mg kg^{-1} or 12.5 mg (Group I) and dose more than 0.2 mg kg^{-1} or 12.5 mg (Group II) of adjuvant intrathecal meperidine in incidence of shivering and secondary outcomes.

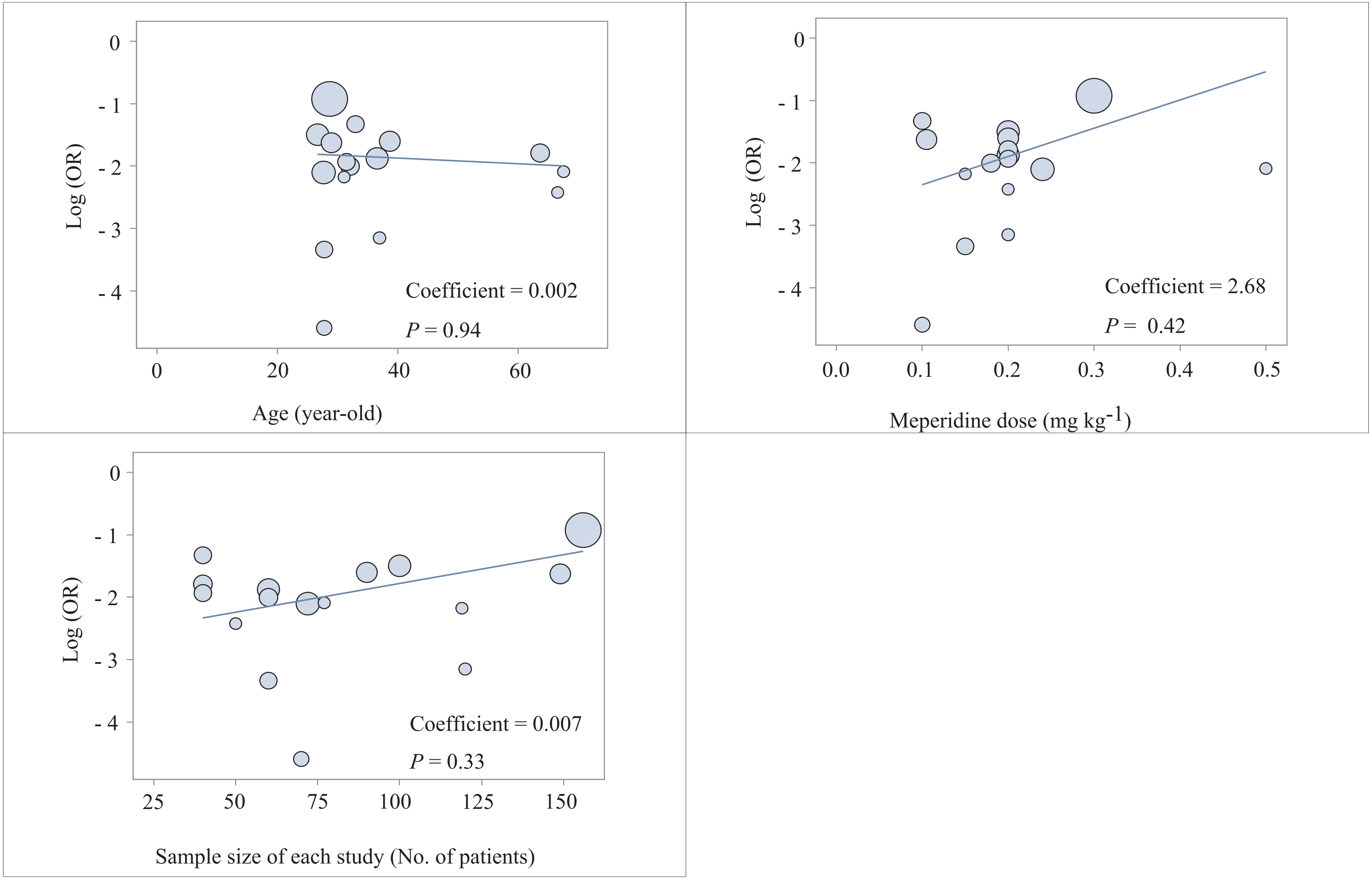

Supplementary Figure 2. Meta-regression of shivering for covariates of age, meperidine dose, and sample size of each study.
